# Finite element analysis of annuloplasty and papillary muscle relocation on a patient-specific mitral regurgitation model

**DOI:** 10.1371/journal.pone.0198331

**Published:** 2018-06-14

**Authors:** Fanwei Kong, Thuy Pham, Caitlin Martin, John Elefteriades, Raymond McKay, Charles Primiano, Wei Sun

**Affiliations:** 1 The Wallace H. Coulter Department of Biomedical Engineering, Georgia Institute of Technology and Emory University, Atlanta, Georgia, United States of America; 2 Aortic Institute of Yale-New Haven Hospital, Yale University, New Haven, Connecticut, United States of America; 3 Cardiology Department, The Hartford Hospital, Hartford, Connecticut, United States of America; Worcester Polytechnic Institute, UNITED STATES

## Abstract

**Objectives:**

Functional mitral regurgitation (FMR) is a significant complication of left ventricle (LV) dysfunction associated with poor prognosis and commonly treated by undersized ring annuloplasty. This study aimed to quantitatively simulate the treatment outcomes and mitral valve (MV) biomechanics following ring annulopalsty and papillary muscle relocation (PMR) procedures for a FMR patient.

**Methods:**

We utilized a validated finite element model of the left heart for a patient with severe FMR and LV dilation from our previous study and simulated virtual ring annuloplasty procedures with various sizes of Edwards Classic and GeoForm annuloplasty rings. The model included detailed geometries of the left ventricle, mitral valve, and chordae tendineae, and incorporated age- and gender- matched nonlinear, anisotropic hyperelastic tissue material properties, and simulated chordal tethering at diastole due to LV dilation.

**Results:**

Ring annuloplasty with either the Classic or GeoForm ring improved leaflet coaptation and increased the total leaflet closing force while increased posterior mitral leaflet (PML) stresses and strains. Classic rings resulted in larger coaptation forces and areas compared to GeoForm rings. The PMR procedure further improved the leaflet coaptation, decreased the PML stress and strain for both ring shapes and all sizes in this patient model.

**Conclusions:**

This study demonstrated that a rigorously developed patient-specific computational model can provide useful insights into annuloplasty repair techniques for the treatment of FMR patients and could potentially serve as a tool to assist in pre-operative planning for MV repair surgical or interventional procedures.

## Introduction

Functional mitral regurgitation (FMR) is a significant complication of left ventricular (LV) dysfunction and is strongly associated with a poor prognosis in patients with heart failure. FMR is commonly treated by using an undersized annuloplasty ring to reduce the septal-lateral diameter of the annulus and improve leaflet coaptation. Annuloplasty is an effective method to correct MR with a low operative mortality rate; however, it remains controversial whether it has a substantial survival benefit over valve replacement or medical treatments for patients with severe FMR [1–3]. Significant recurrence of MR has been reported following annuloplasty [3–5]. The use of undersized annuloplasty may augment posterior leaflet tethering which contributes to persistent MR following surgical annuloplasty [6].

The three-dimensional (3D)-shaped GeoForm ring was specifically designed to aggressively reduce the septal-lateral distance while displacing the posterior annulus towards the left atrium, which may counteract the tethering forces of the chordae [7]. Papillary muscle relocation (PMR) as an adjunct procedure to downsized ring annuloplasty was developed to reduce leaflet tethering, tenting area, and coaptation depth by pulling the papillary muscle tips closer to the annulus[8]. Both techniques have demonstrated potential in effectively relieving MR and preventing recurrent MR, although cases of recurrent MR still occur [7,8].

The finite element method (FEM) has been developed and utilized as a possible tool for evaluating clinical treatment strategies and predicting treatment outcomes. While several studies have applied FEM to study the effect of annuloplasty on treating MR using various ring shapes [9–15], to our knowledge, no study has used human MR models to investigate the effect of PMR on MV biomechanics [16]. Due to the complexity of the MV apparatus, many limitations continue to undermine the accuracy of computational analysis, such as the use of simplified chordal morphology [9,12,13,15], simplified leaflet geometries [10–12,14], and assumed stress-free initial geometries. Also, such models did not account for the presence of leaflet tethering associated with FMR with LV dilation. Recently, we presented a case study of an FMR patient where the leaflet tethering force were accounted for in the reference geometries, which is essential for accurate MV modeling and quantification of leaflet coaptation and stress distribution [17,18].

In this study, we evaluated ring annuloplasty and PMR treatments in the same FMR patient using computational modeling. We extended the model to include the LV component, which allowed us to simulate the PM motions associated with annuloplasty procedures. The goals were to: a) predict the outcomes of ring annuloplasty treatments using Edwards Classic and GeoForm rings on this specific MR patient, and b) study the effects of ring sizes and PM relocation on MV coaptation and biomechanics. Computational models of MR repair with five sizes of the Classic ring and two sizes of the GeoForm ring, with and without PMR procedures, were developed to simulate the deformed MV geometries, leaflet closing force, and leaflet stress and strain post-repair for the patient.

## Methods

### Patient-specific model geometry

Full-phase cardiac multi-slice Computed Tomography (MSCT) scans of a 71-year-old male patient were collected from Hartford Hospital (Hartford, CT). The use of de-identified patient image data for this study was approved by the Institutional Review Board at the Hartford Hospital (Hartford, CT) and the Georgia Institute of Technology. Details of patient information were described in a previous publication(17). Briefly, the patient was referred for transcatheter aortic valve replacement (TAVR) due to severe aortic stenosis and was also diagnosed with severe MR from echocardiogram images. The LV chamber was dilated with severe regional global hypokinesis, such that the inferior wall was akinetic. The posterior leaflets were tethered and restricted in motion.

**Fig 1:**
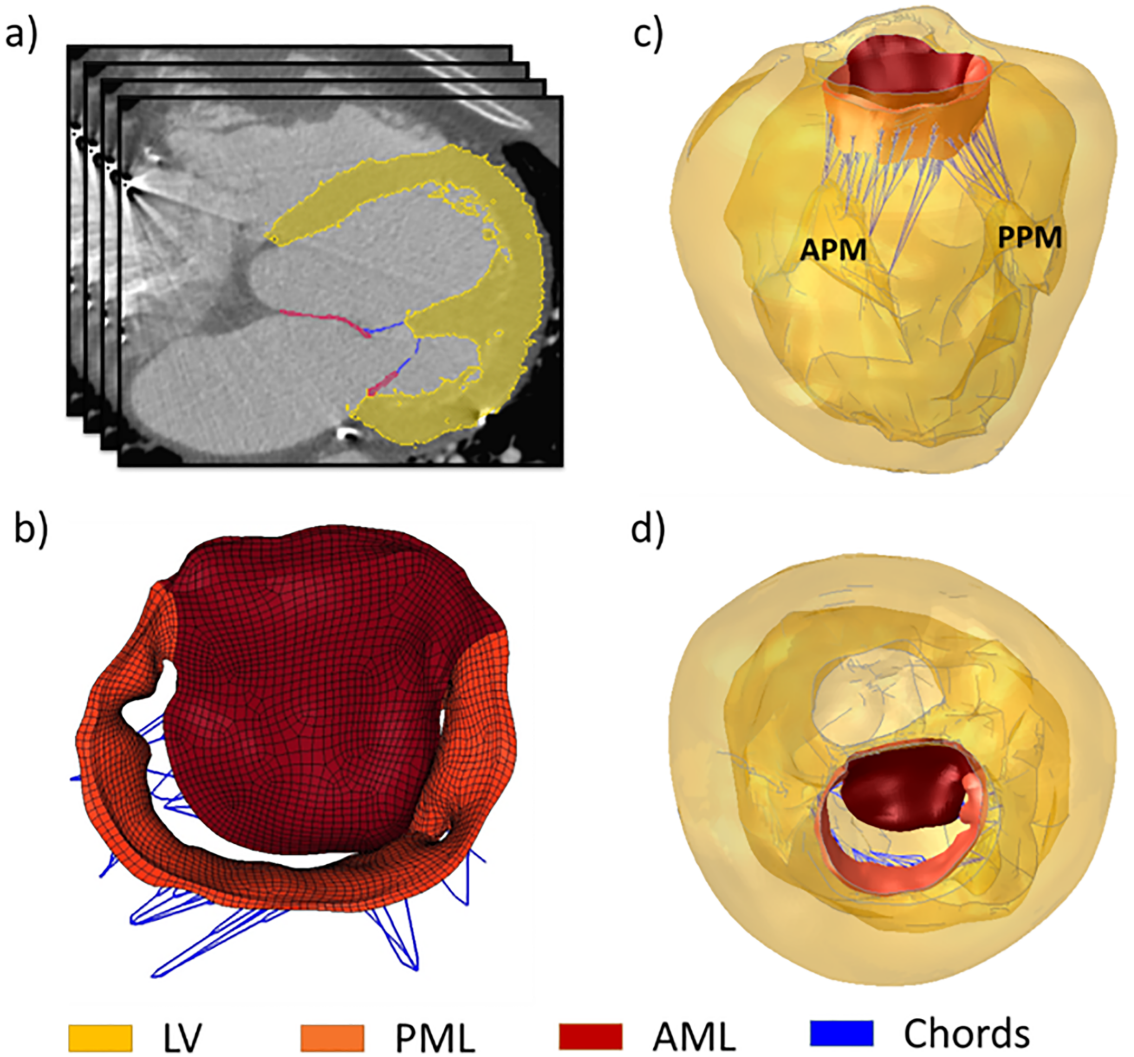
Construction of FE model from patient-specific MSCT images. a) Segmentation of model geometries on MSCT images, b) FE model of MV c) side view and d) top view of FE MV model with LV attached.

For this patient, the FE model containing the anterior mitral leaflet (AML), posterior mitral leaflet (PML) and chordae tendineae (chords) was constructed from and validated against MSCT images(17). Furthermore, detailed geometries of the left ventricle (LV) and papillary muscles (PM) were identified and segmented from MSCT images at middle diastole using Avizo (Version 8.0, Burlington, MA) (Fig 1a). The surface geometries of the LV and PM were then imported into HyperMesh (Altair Engineering, Inc., MI) to generate a 3D mesh which was combined with the previous MV model (Fig 1b–1d).

### Constitutive modeling of mitral tissues

An anisotropic hyperelastic material model, based on Holzapfel *et al*. [19,20], was adopted to characterize the mechanical behaviors of the mitral leaflet tissues. The isotropic hyperelastic Ogden material model was used to characterize the mechanical properties of the chords. The material properties of the MV apparatus and LV were obtained by fitting in-house biaxial test data of an age- and gender-matched human cadaver heart, i.e., a 69-year-old male patient. Details on the implementation of the model and material parameters of the MV apparatus were previously published [17]. Briefly, the mitral tissues are assumed to be composed of a matrix material with two families of embedded fibers, each with a preferred direction. The fiber directions can be mathematically described using two-unit vectors. The strain invariant, I_1_, describes the matrix material, and the strain invariant, I_4i_, describes the properties of the fiber families and is equal to the square of the stretch in fiber direction, *i*. The strain energy function *W* can be expressed as

$$\text{W}={\text{C}}_{10}\left\{\text{exp}\left[{\text{C}}_{01}\left(\text{I}\prime_{1}-3\right)\right]-1\right\}+\frac{{\text{k}}_{1}}{2{\text{k}}_{2}}\sum_{\text{i}=1}^{2}\left[\text{exp}\left\{{\text{k}}_{2}{\left[\kappa \text{I}\prime_{1}+\left(1-3\kappa \right)\text{I}\prime_{4\text{i}}-1\right]}^{2}\right\}-1\right]+\frac{1}{\text{D}}{\left(\text{J}-1\right)}^{2},\text{i}=1,2$$ (1)

where, C_10_, C_01_, k_1_, k_2_ and D are material constants. Additionally, a dispersion parameter κ describes the distribution of fiber orientation. The parameters for the LV are as follows: ρ = 1.1 g/cm3, C_01_ = 10.678, C_10_ = 0.034 kPa, k_1_ = 3.903 kPa, k_2_ = 26.900, κ = 2.427E-8, θ = 29.479 degrees.

### Boundary and loading conditions

The MV annuloplasty and PMR procedures were simulated in three steps: pretension, annuloplasty, and PMR, described below. All simulations were performed in ABAQUS Explicit (Version 13). In summary, displacement boundary conditions were applied on the chordal origins to simulate pretension in the first step. For annuloplasty, displacement boundary conditions were applied on the annulus nodes to simulate annulus deformations; boundary conditions were released on chordal origins to simulate the PM motions resulted from annuloplasty while point-loads were applied to maintain the reaction forces resulted from pre-tension. For PMR, displacement boundary conditions were applied on the chordae origins on APM to simulate relocation of APM towards left trigone; annulus nodes and other chordae origins were fixed in place. After both the annuloplasty and PMR procedures, a transvavular pressure of 114 mmHg was applied to the mitral valve to simulate valve closure. These conditions were described in detail in the following subsections.

#### Pretension simulation

**Fig 2:**
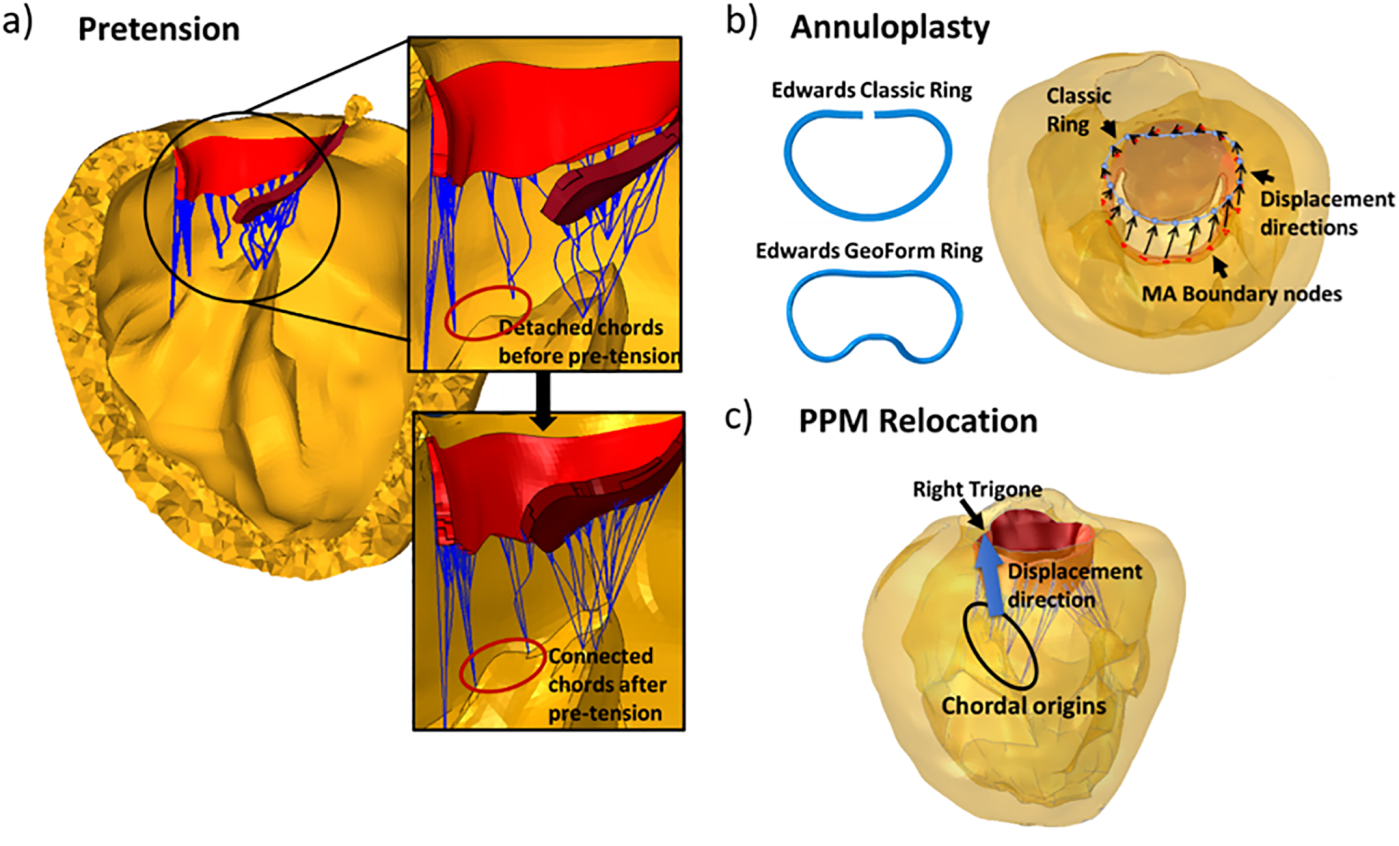
Illustration of the modeling process. a) the pretension simulation steps b) Edwards Classic and GeoForm ring geometries in 2D and the superior view of the left heart showing MA boundary nodes and c) PMR simulation steps.

To simulate the tethering of the PML, posterior chords originating from a total of 8 chordal origins were shortened by translating the chordal origins towards the annulus plane along the original axes of the chords[17]. In the first step of the simulation, those chordal origins were displaced to their original locations to generate the tethering tension within the chords (Fig 2a). After displacing the chordal origins to their original locations on the PPMs, a “rough” contact (infinite coefficient of friction between the contact surfaces, defined in ABAQUS) with no separation behavior was enforced to connect the chords with the LV. The reaction force at each chordal origin was output at the end of this step.

#### Annuloplasty ring implantation simulation

Ring annuloplasty procedures with the Edwards Classic and GeoForm annuloplasty rings (Edwards Lifesciences, Irvine, California) were simulated in this study. A common technique to determine an appropriate ring size is to use a ‘sizer’ which gauges the native mitral annulus (MA) size For this patient, the appropriate ring sizes for Classic and GeoForm rings were determined by two different methods following the respective surgical procedures for Classic ring and GeoForm ring implantation [7,21]. For the Classic ring, a ring size of 38 is chosen based on the similarity between the surface area of the device and the surface area of the AML. For the GeoForm ring, a ring size of 38 was chosen based on the patient’s intertrigonal distance. In addition, Classic rings of sizes 32, 34, 36 and 40 and a GeoForm ring with size 36 were simulated to evaluate the effect of downsizing and annulus reshaping.

To align the virtual ring with the patient MA plane, least-square planes were created for both the ring and the patient MA. The ring was rotated until its least square plane was parallel to that of patient MA. Then middle anterior and posterior portions of the ring were aligned with the middle anterior and posterior portions of the MA and the ring was positioned such that the anterior portion of the ring overlap with the anterior MA as much as possible to avoid excessive displacement of aortic opening due to ring implantation.

Following ring alignment, a total of 18 node clusters uniformly distributed along the patient MA were identified as MA boundary nodes; each cluster contained 3 adjacent nodes. On the virtual ring, 18 uniformly distributed nodes were also identified. In the simulations, suturing the annuloplasty ring to the MA was simulated by imposing kinematic displacements on the 18 node clusters on the MA from their original locations to the locations of the 18 corresponding nodes identified on the virtual ring (Fig 2b). During this step, the boundary conditions on the chordal origins were unfixed to allow PPM movements due to the ring implantation. Meanwhile, a point load equivalent to the reaction force, obtained at each chordal origin from the Pretension Simulation, was applied to the same chordal origin to account for the tension within the chords due to leaflet tethering at diastole.

To simulate MV closure during systole following the virtual ring implantation, the chordal origin nodes were displaced from middle diastole to middle systole based on their dynamic motion tracked from MSCT images [17]. A pressure load of 114 mmHg was applied on the ventricular surfaces of AML and PML to simulate the systolic transvalvular pressure.

#### Papillary muscle relocation (PMR) simulation

During the clinical PMR procedure, the head of the anterior papillary muscle (APM) is relocated anteriorly by passing a suture through the fibrous portion of the APM tip and then to the mitral annulus at the left trigone [8,22]. The PMR simulation steps were constructed in a similar manner. Briefly, following the virtual annuloplasty ring implantation, chordal origins of the APM were displaced 5mm anteriorly and towards the lateral trigone. (Fig 2c). The annular geometry was fixed during this step to maintain the ring shape. A transvalvular pressure of 114 mmHg was applied to simulate the MV closure after PMR simulation.

### Data analysis

To assess the impact of each procedure on MR, the coaptation depth and length, intercommissural distance, septal-lateral distance, regurgitant orifice area, and tenting area at middle systole were measured from the simulation results as described in our previous study [17]. To study the changes in LV geometry in response to ring annuloplasty and PMR treatments, the APM-MA distance and inter-PM distance were also measured. The APM-MA distance was defined as the distance between the anterior papillary muscle tip and the least-square plane of the MA. The average maximum principal stress and strain of the midsections of the AML and PML (a 10 mm by 10 mm square region on AML and a 9 mm by 9 mm square region on PML), and leaflet contact forces were output from simulations.

## Results

### Deformed model geometries

#### Mitral valve

After ring annuloplasty (Table 1), the largest reduction in the septal-lateral distance was 55% for the GeoForm ring size 36 and the largest reductions in annular area and intercommissural distance were 13% and 37% respectively for the Classic ring size 32.

**Table 1:** Measurements of intercommissural distance, septal-lateral distance and annular area of patient MV geometry at systole and FE geometries after annuloplasty simulations.

	Intercommissural distance (mm)	Percentage reduction in Intercommissural distance (%)	Septal-lateral Distance (mm)	Percentage reduction in Septal-lateral Distance (%)	Annulus Area (mm^2)	Percentage reduction in Annulus Area (%)
**Patient MV**	39.1		33.51		944.693	
**Classic 32**	33.94	13.20	20.41	39.10	594.35	37.09
**Classic 34**	35.46	9.32	21.97	34.44	659.47	30.19
**Classic 36**	37.16	4.96	23.50	29.88	731.20	22.60
**Classic 38**	38.68	1.08	24.91	25.65	811.67	14.08
**Classic 40**	40.92	-4.64	26.52	20.85	892.67	5.51
**GeoForm 36**	36.34	7.07	15.09	54.97	612.61	35.15
**GeoForm 38**	38.20	2.31	16.27	51.44	682.57	27.75

**Fig 3:**
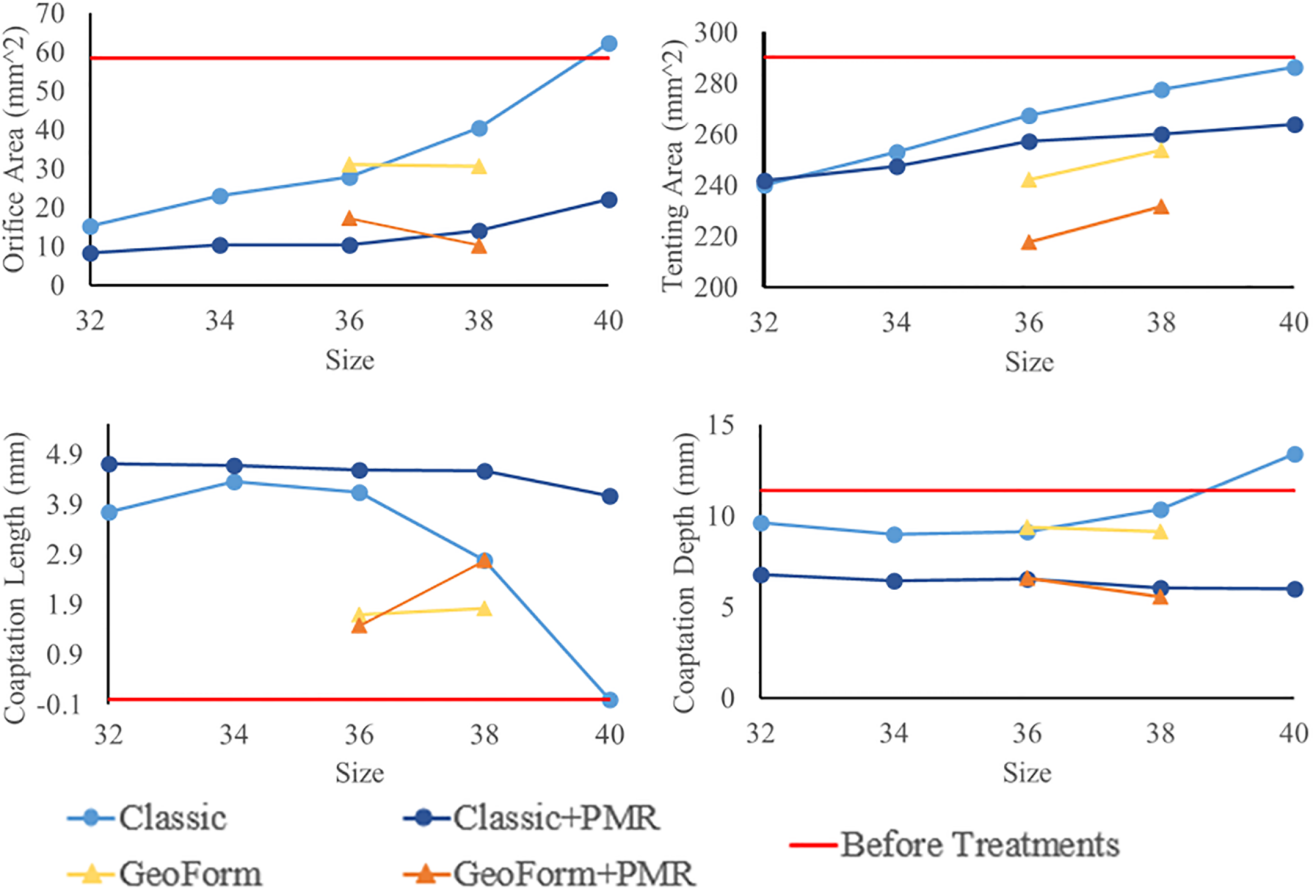
Orifice area, tenting area, coaptation depth and length measurements of deformed MV geometries after ring annuloplasty and PMR simulations for all ring sizes and shapes.

The regurgitant orifice area, leaflet tenting area, and coaptation length and depth at A2 and P2 sections for each case are shown in Fig 3. Compared to the patient MV prior to treatment, the size 38 Classic ring reduced the regurgitant area, tenting area, and coaptation depth by 30%, 17%, and 9%, respectively, while the size 38 GeoForm ring led to even greater reduction in the regurgitant area, tenting area, and coaptation depth (47%, 26%, and 20%, respectively). For the Classic rings, downsizing resulted in smaller regurgitant areas and leaflet tenting areas. The leaflet coaptation length increased and coaptation depth decreased with decreasing ring sizes from size 40 to 34, while the smallest size 32 did not further improve the coaptation length. On the contrary, smaller GeoForm rings did not produce smaller regurgitant areas or reduced tenting areas and coaptation lengths. PMR in adjunct to ring annuloplasty further reduced the regurgitant orifice areas and leaflet tenting areas and increased leaflet coaptation lengths and decreased coaptation depths for both ring types and all sizes.

**Fig 4:**
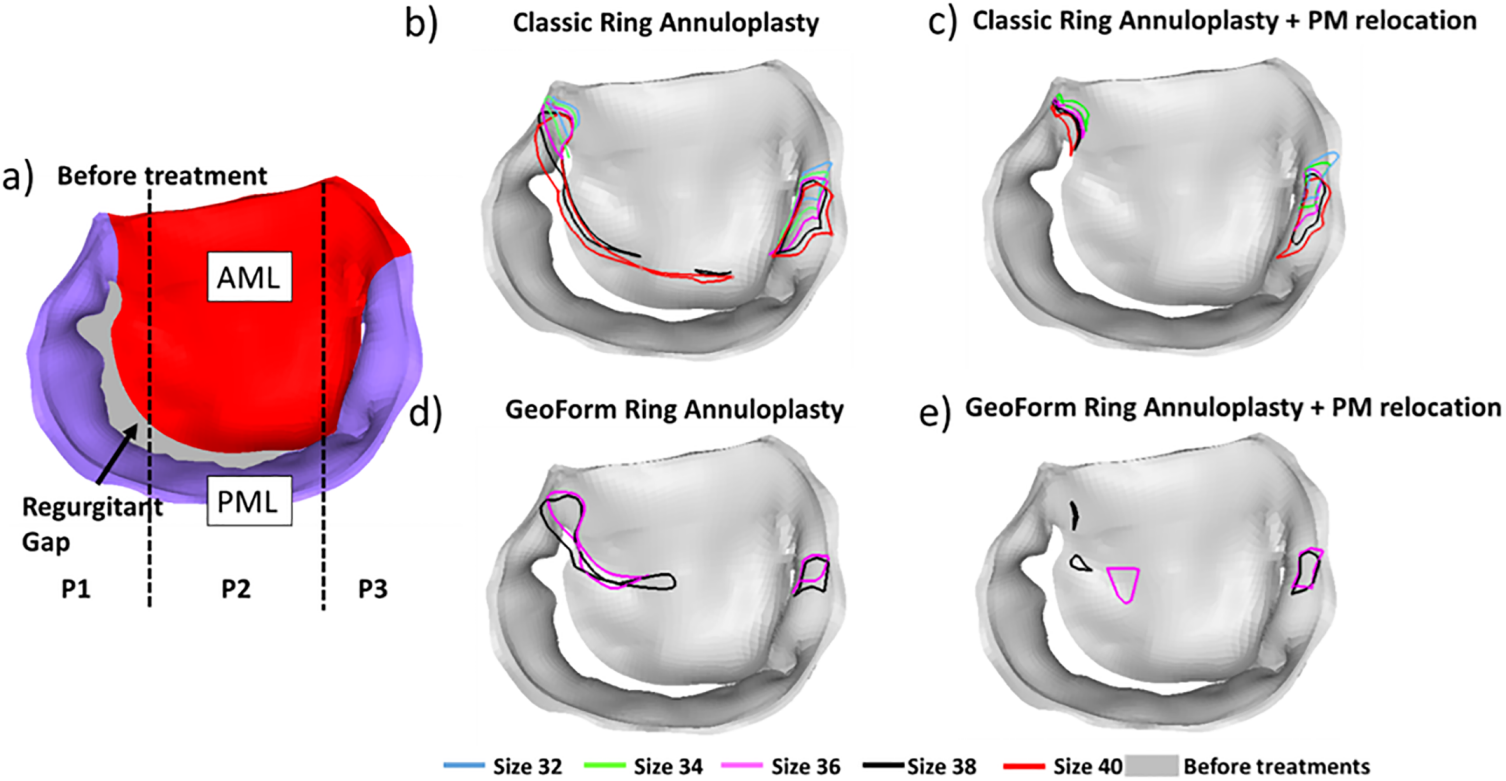
2D orifice area of deformed MV at systole. a) patient MV before annuloplasty, b) Classic ring annuloplasty, c) Classic ring annuloplasty with PMR, d) GeoForm ring annuloplasty and e) GeoForm ring annuloplasty with PMR.

The 2D projections of the regurgitant areas are shown in Fig 4. Before any treatments, the orifice gap for this patient was on the posterior lateral portion of the annulus, spanning from P1 to the middle P2 sections of the posterior leaflet (Fig 4a). Following annuloplasty with either the size 38 Classic or GeoForm ring, coaptation was improved at the P2 location while the orifice gaps at the lateral commissure at P1 persisted and some were newly formed at the septal commissure of P3. When smaller size rings were used, the regurgitant orifice areas decreased at both P1 and P3 but were still present even when the smallest ring size was used (Fig 4b and 4d).

By performing PMR, the regurgitant orifice gap at the lateral commissure was greatly reduced for all Classic rings and was eliminated for the smallest ring of size 32 (Fig 4c). For GeoForm rings, PMR greatly reduced the regurgitant orifice gaps at the lateral commissure for both size 36 and 38 rings (Fig 4e). However, for the GeoForm ring size 36, an orifice gap was newly formed near P2 after PMR, as shown in Fig 4e.

#### Left ventricle

**Fig 5:**
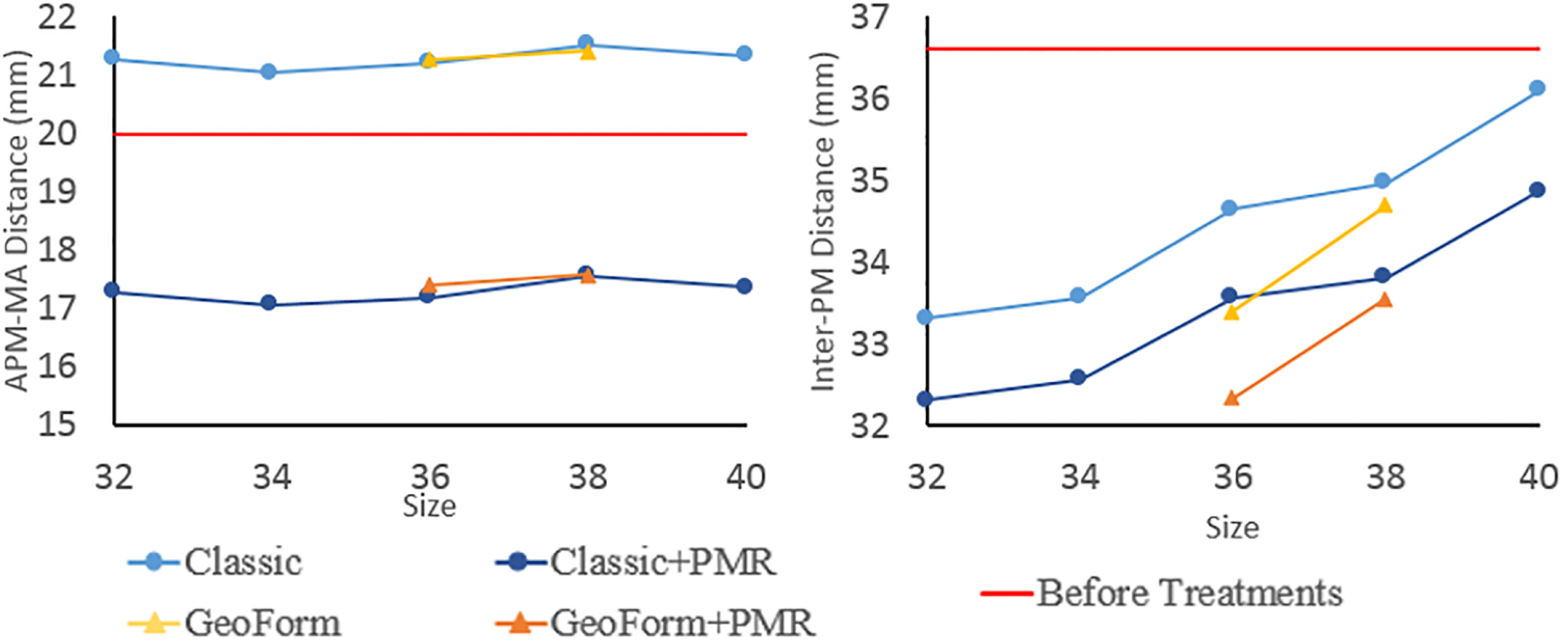
Inter-PM and PM-MA distances of deformed MV geometries after ring annuloplasty and PMR simulations for all ring sizes and shapes.

Incorporating the LV in the MV model allowed for quantitative analysis of the changes to the inter-PM distance and APM-MA distance after ring annuloplasty and PMR treatments. Fig 5 compares the APM-MA and inter-PM distances from all simulations. Following annuloplasty with the size 38 Classic ring, the APM-MA distance increased by 8% and the inter-PM distance decreased by 4%. For the GeoForm ring of size 38, the APM-MA distance increased by 7% and the inter-PM distance decreased by 8%. Ring annuloplasty increased the APM-MA distance for all cases while ring downsizing reduced the inter-PM distances for both Classic and GeoForm rings. Subsequent PMR further reduced both inter-PM and PM-MA distances for all cases.

### Leaflet stress and strain

**Fig 6:**
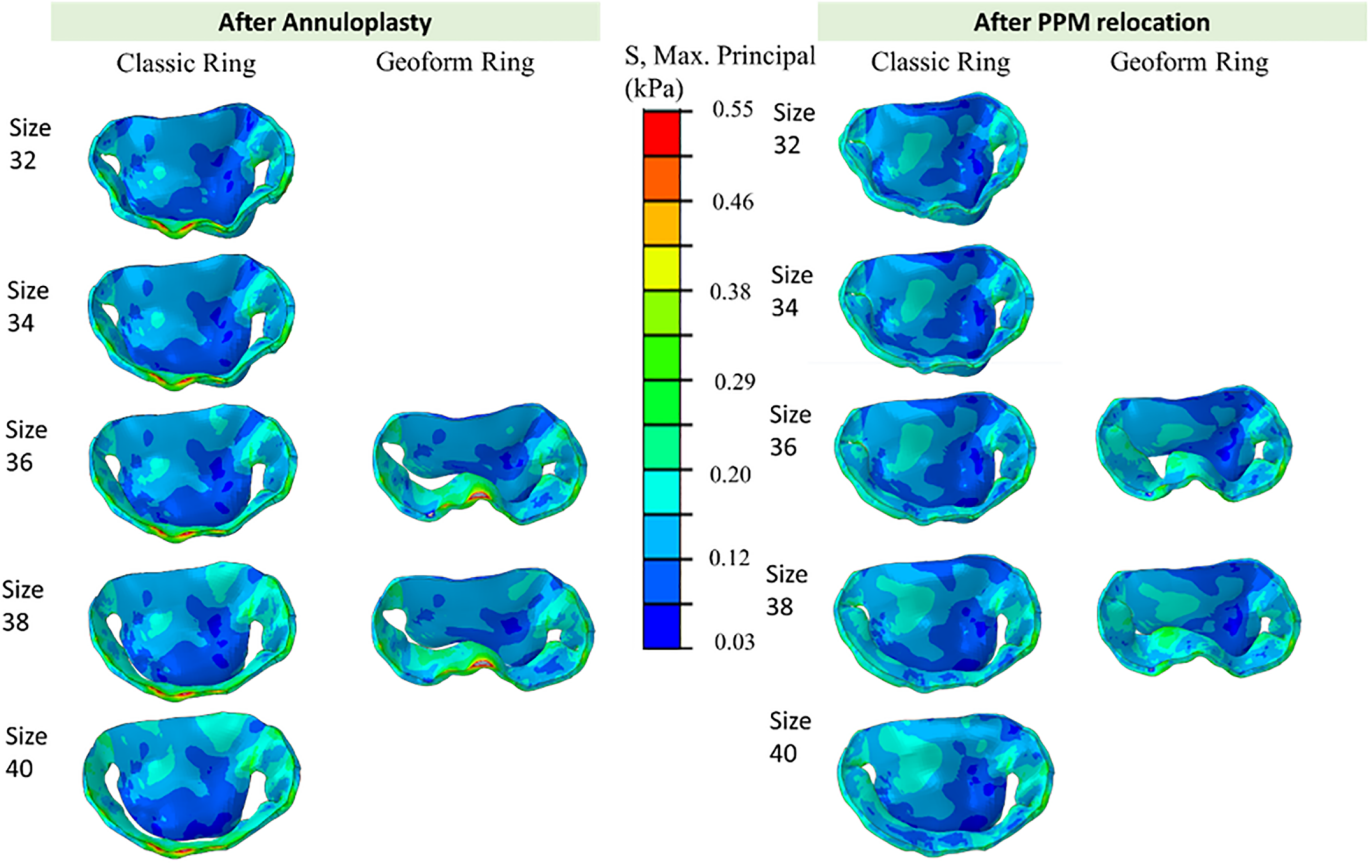
Maximum principal strain distribution after annuloplasty (left panel) and additional PMR for Classic and GeoForm rings with different sizes.

**Fig 7:**
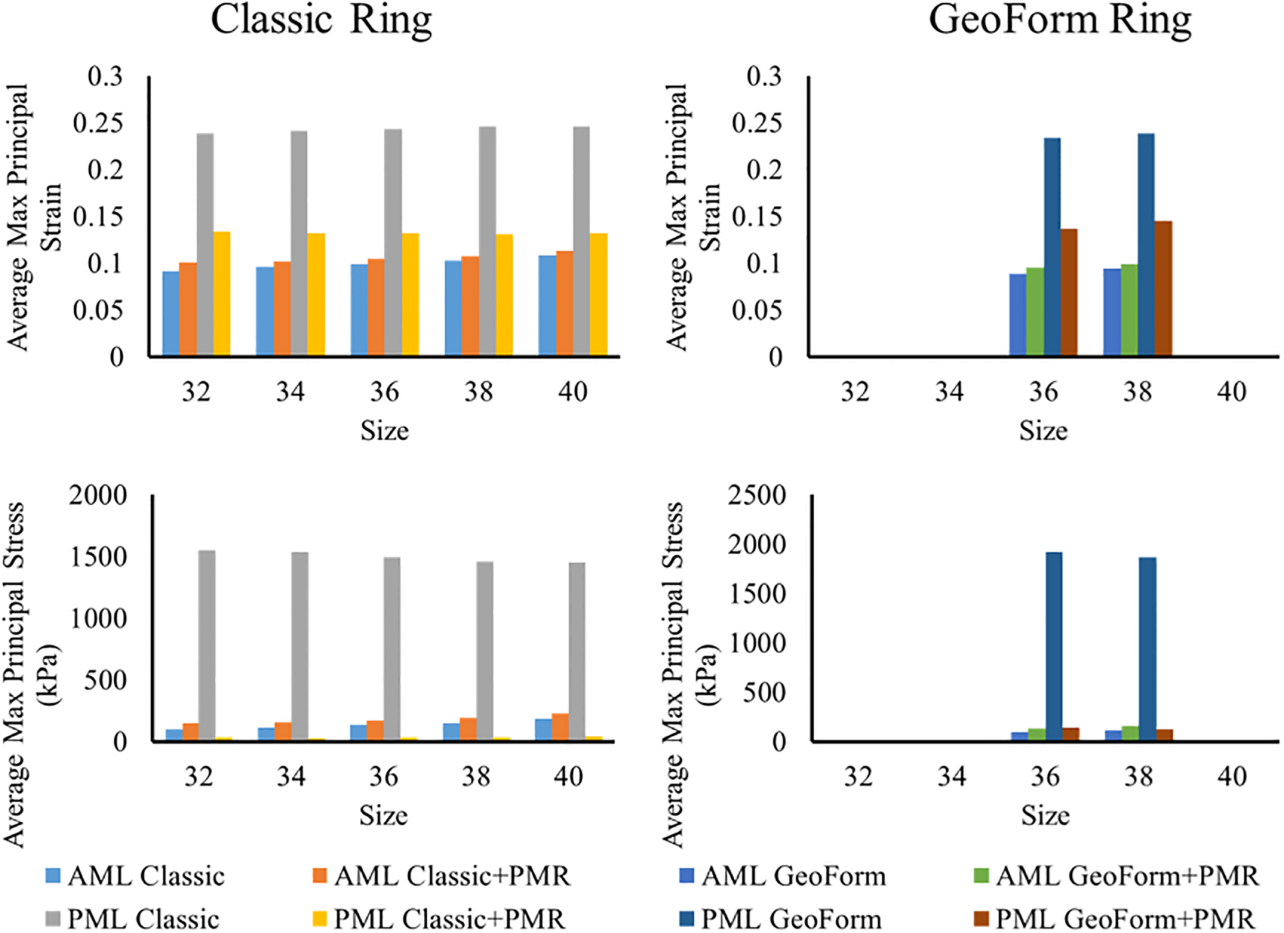
Median maximum principal stress and median maximum principal strain of AML and PML for deformed FE models after Classic ring (left panel) and GeoForm ring (right panel) annuloplasty and PMR.

Fig 6 displays the maximum principal strain distribution on the MV leaflets following ring annuloplasty and PMR. High strain was observed near the annulus at the P2 section of PML in all cases. Ring annuloplasty with Classic ring size 38 increased the average stress and strain of the PML by 1836% and 68% respectively, and annuloplasty with GeoForm ring size 38, increased the average stress and strain by 2377% and 63% respectively. However, the average stress and strain of AML decreased by 11% and 18% for Classic ring size 38 and by 18% and 36% for GeoForm ring size 38. Fig 7 summarizes the average stress and strain values for all cases. A trend of decreasing stress and strain on the AML was observed with smaller ring sizes but was not evident for the PML. GeoForm rings caused higher maximum principal stress values on the PML compared to the same size Classic rings. PMR greatly reduced the stress and strain on the PML for all ring sizes and shapes while slightly increasing the stress and strain on the AML.

### Leaflet contact force

**Fig 8:**
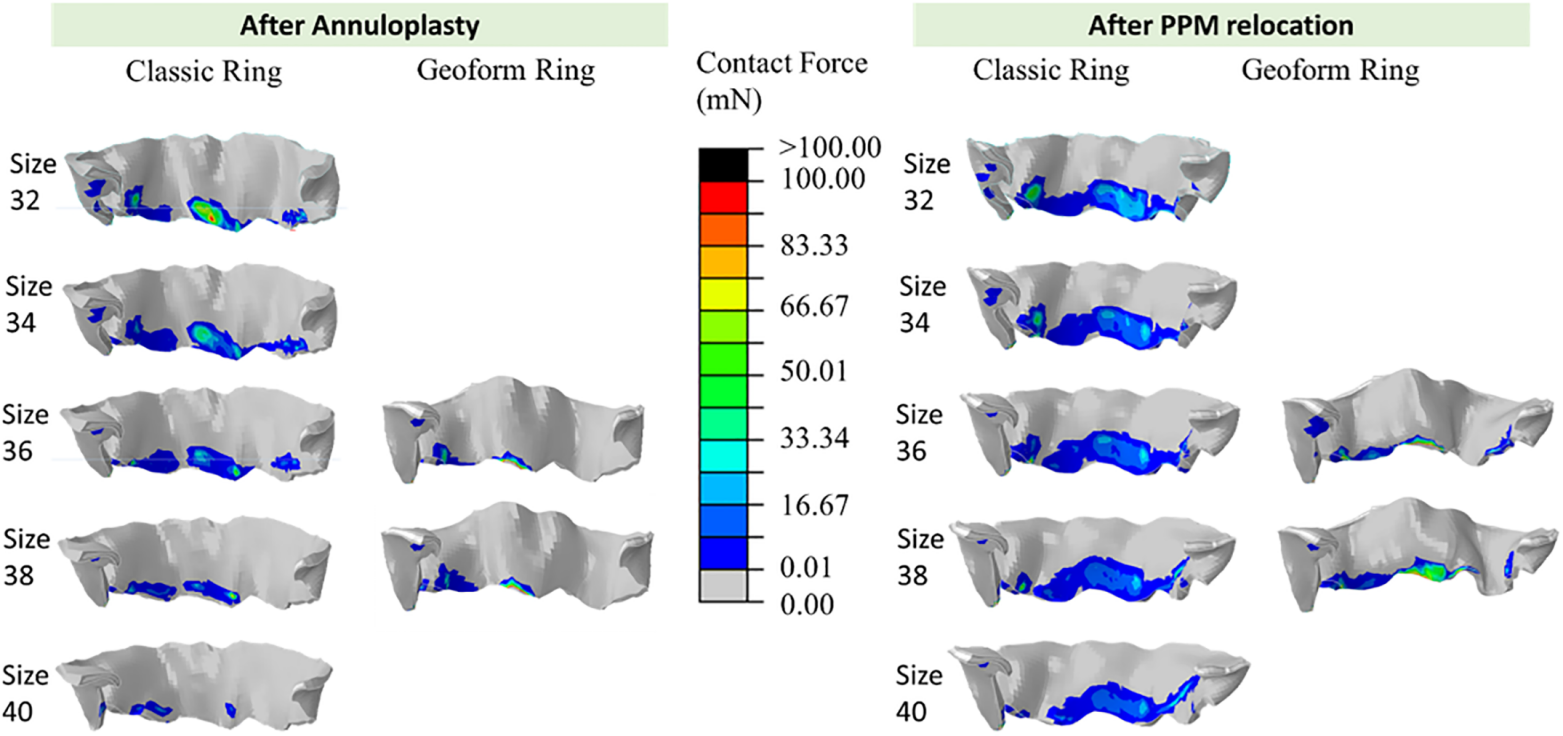
Contact force distribution after annuloplasty (left panel) and after PMR in addition to annuloplasty (right panel) for Classic and GeoForm rings.

Due to severe MR, this patient had little leaflet coaptation during systole prior to MR repair. After ring annuloplasty with Classic ring size 38, the total leaflet contact area and force increased to 41 mm^2^ and 0.5N. With GeoForm ring size 38, the total leaflet contact area and force increased to 32 mm^2^ and 0.6N. Fig 8 displays the contact force distribution after annuloplasty and after PMR in addition to annuloplasty for Classic and GeoForm rings. Downsizing with Classic rings increased the leaflet contact area and force. The total contact area and force with the smallest Classic ring size 32 increased by 480% and 1317% compared to those of the largest ring size. However, downsizing with a smaller GeoFrom ring decreased the contact area by 27%, with no evident change in total contact force. PMR increased the total contact area and force for all sizes. For the Classic rings, compared to ring annuloplasty alone, PMR increase the total contact area and force by 262% and 244% on average, respectively. PMR with the GeoForm rings increased the total contact force and area by 205% and 170% on average, respectively.

## Discussion

Patient-specific FE modeling of MR conditions could provide useful insights into the mitral valve biomechanics and potentially serve as a tool to assist in accurate and quantitative pre-operative planning for MV repair surgeries. This study developed computational models to simulate the treatment outcomes of ring annuloplasty with Classic and GeoForm rings for an MR patient, and further studied the effect of using downsized Classic and GeoForm rings and applying PMR on MV biomechanics and leaflet coaptation. The simulation results demonstrated that ring annuloplasty with Classic and GeoForm rings improved leaflet coaptation and increased the leaflet total closing force while increased the PML stress and strain. Ring downsizing further improved leaflet coaptation and leaflet total closing force and decreased the stress and strain on the AML. Higher coaptation forces and larger coaptation areas were observed with Classic rings. Adjunct PMR procedures can improve leaflet coaptation, decrease stress and strain in the PML, and improve coaptation force and area for both ring shapes and all sizes.

FMR is often treated with an annuloplasty ring that is one or two sizes smaller than the actual MA to ensure sufficient leaflet coaptation [23]. For this patient, using the actual size determined by the MA dimensions (Classic ring size 38), the simulation demonstrated improved leaflet coaptation but with a residual lateral regurgitant orifice. By downsizing the ring by one to two sizes, the lateral regurgitant gap was largely eliminated with only observable gaps near the two commissures, consistent with clinical observations. It is worth noting that after ring annuloplasty, a new regurgitant gap appeared at the right commissure. Using a different ring shape with a higher curvature at the anterior annulus may reduce the regurgitant gap in this case, by pulling in the annulus at this location.

Clinical and computational results have demonstrated that implanting the GeoForm ring is effective for improving coaptation with a low rate of recurrent IMR [7,13,15,24], and counteracting PML tethering by lifting the P2 region of the PML towards the left atrium [7]. For this patient, however, although the aggressive reduction in septal-lateral diameter with a GeoForm ring greatly reduced the leaflet tenting area, the coaptation length was not greatly improved compared to the Classic rings. For this patient, once the posterior annulus was lifted, the PML was too short in length and could barely contact the free edge region of the AML, resulting in insufficient coaptation.

Recurrent MR following restricted annuloplasty in FMR patients has been considered to associated with augmented the posterior leaflet tethering and restricted PML motion [7,25]. Including LV in the simulation was necessary in understanding the resulted posterior leaflet tethering following treatments. For this patient with PML tethering, we observed a substantial increase in stress in the PML after GeoForm and Classic ring annuloplasty, likely resulted from the increase in APM-MA distance. However, increase in the APM-MA distance or augmented PML tethering due to downsizing was not evident for either the Classic or GeoForm ring. Continuous remodeling of the leaflets (e.g., elongation of the PML) and the LV after the annuloplasty may reduce the high stress seen in the PML.

Applying PMR improved leaflet coaptation and reduced the leaflet strain and stress for all ring sizes and both ring shapes in this study. PMR as an adjunct to ring annuloplasty has demonstrated promise in preventing recurrent MR for FMR annuloplasty treatments, and can considerably decrease the MV tenting area and coaptation depth, consistent with clinical findings [8]. Applying adjunct PMR was more effective than using a downsized ring: PMR with the Classic size 38 ring produced better coaptation than using the smallest Classic ring alone, in terms of regurgitant area, tenting area, and coaptation length and depth. Regarding stress and strain on the tethered PML and posterior annulus, applying PMR effectively relieved the high stress and strain for both ring shapes and all sizes. Compared with ring annuloplasty alone, PMR increased the total leaflet contact force and contact area but did not increase the maximum contact force, indicating that PMR induced more a uniform contact force distribution over a larger contact area. Since this patient had a posterior lateral regurgitant gap and PML tethering near the anterior PM, only the anterior PM was displaced towards the left trigone to reduced PML tethering. Displacing the posterior PM towards the right trigone at the same time has been conducted clinically [8] and could help eliminate the regurgitant gap that appeared at the right commissure.

## Limitations

Ring annuloplasty procedures were simplified as prescribed boundary conditions on the annulus rather than implanting a virtual ring with its full cross-sectional geometry and material properties. MA motion was constrained during the systolic simulation step; thus, the ring motion was eliminated and the final ring locations at systole were assumed. Since this patient did not undergo MV repair treatments, the post-treatment systolic motions of the PMs were not available and were therefore assumed to be unchanged from before treatments. However, since identical simplifications were applied on all simulations, the study provided valuable insights in comparing the effect on different ring shapes, ring sizes, and PMR procedures on the treatment outcomes and MV biomechanics. Although the leaflet and chordae tendineae materials were age- and gender-matched, they were not determined from the same MR patient. Material properties determined inversely from CT images would be ideal to obtain accurate simulation results. Furthermore, our model did not include hemodynamic effects imposed by the blood flow. Fluid-structural-interaction models of MR patients will be applied in the future to investigate the changes in systolic regurgitation due to ring annuloplasty and PMR procedures. Furthermore, the simulation results were not validated against the clinical data following the annuloplasty or PMR treatments, since the patient studied underwent neither of the treatments. However, the simulation results still provide understanding of treatment outcomes and MV biomechanics related to the annuloplasty ring size and shape as well as the adjunct PMR treatments, since all the simulations were performed using the same model.

## Supporting information

S1 Table Simulation results after annuloplasty (left) procedures and after PMR procedures (right) for both ring shapes and all ring sizes. The results include orifice area (mm^2^), coaptation area (mm^2^), coaptation depth (mm), tenting area (mm^2^), APM-MA distance (mm), inter-PM distance (mm), average maximum principal strain and stress (kPa) for AML and PML, total contact force (mN) and total contact area (mm^2^).
(XLSX)
